# Prognostic value of mid-regional pro-adrenomedullin (MR-proADM) in patients with community-acquired pneumonia: a systematic review and meta-analysis

**DOI:** 10.1186/s12879-016-1566-3

**Published:** 2016-05-26

**Authors:** Dan Liu, Lixin Xie, Haiyan Zhao, Xueyao Liu, Jie Cao

**Affiliations:** Department of Respiratory Medicine, Tianjin Medical University General Hospital, Tianjin, 300070 China; Medical School, Nankai University, 94 Weijin Road, Tianjin, 300071 China; Department of Pulmonary & Critical Care Medicine, Chinese PLA General Hospital, 28 Fuxing Road, Beijing, 100853 China

**Keywords:** MR-ProADM, CAP, Prognosis, Meta-analysis

## Abstract

**Background:**

The early identification of patients at risk of dying from community-acquired pneumonia (CAP) is critical for their treatment and for defining hospital resource consumption. Mid-regional pro-adrenomedullin (MR-proADM) has been extensively investigated for its prognostic value in CAP. However, the results are conflicting. The purpose of the present meta-analysis was to explore the diagnostic accuracy of MR-proADM for predicting mortality in patients suffering from CAP, particularly emergency department (ED) patients.

**Method:**

We systematically searched the PubMed, Embase, Web of Knowledge and Cochrane databases. Studies were included if a 2 × 2 contingency table could be constructed based on both the MR-proADM level and the complications or mortality of patients diagnosed with CAP. The prognostic accuracy of MR-proADM in CAP was assessed using the bivariate meta-analysis model. We used the Q-test and *I*^2^ index to evaluate heterogeneity.

**Results:**

MR-proADM displayed moderate diagnostic accuracy for predicting complications in CAP, with an overall area under the SROC curve (AUC) of 0.74 (95 % CI: 0.70–0.78). Eight studies with a total of 4119 patients in the emergency department (ED) were included. An elevated MR-proADM level was associated with increased risk of death from CAP (RR 6.16, 95 % CI 4.71–8.06); the *I*^*2*^ value was 0.0 %, and a fixed-effects model was used to pool RR. The pooled sensitivity and specificity were 0.74 (95 % CI: 0.67–0.79) and 0.73 (95 % CI: 0.70–0.77), respectively. The positive likelihood ratio (PLR) and negative likelihood ratio (NLR) were 2.8 (95 % CI, 2.3–3.3) and 0.36 (95 % CI, 0.29–0.45), respectively. In addition, the diagnostic odds ratio (DOR) was 8 (95 % CI, 5–11), and the overall area under the SROC curve was 0.76 (95 % CI, 0.72–0.80).

**Conclusions:**

Our study has demonstrated that MR-proADM is predictive of increased complications and higher mortality rates in patients suffering from CAP. Future studies are warranted to determine the prognostic accuracy of MR-proADM in conjunction with severity scores or other biomarkers and to determine an optimal cut-off level.

**Electronic supplementary material:**

The online version of this article (doi:10.1186/s12879-016-1566-3) contains supplementary material, which is available to authorized users.

## Background

Lung infections are the most frequent type of infection worldwide. Community-acquired pneumonia (CAP) is a disease with a very wide range of possible outcomes. A considerable proportion of patients can be treated as outpatients. Additionally, CAP may serve as a sepsis precursor and is more likely to result in death in critically ill patients [1]. Risk stratification is crucial to CAP patient management in the emergency department (ED) to select the most appropriate care setting, including outpatient treatment, admission to a hospital ward (HW) or admission to an intensive care unit (ICU). Thus, clinical studies are currently focusing on searching for the most appropriate prognostic factors and risk stratification tools in respiratory medicine.

Several risk scores (PSI, CURB65) have been developed for assessing the severity of CAP and predicting mortality [2, 3]. However, none are ideal for clinical use. Some scores are too complicated to use in daily practice, and some are not exempt from false-positive and false-negative results. Blood biomarkers (for example, C-reactive protein, procalcitonin, soluble triggering receptor expressed on myeloid cells-1, and interleukin-1 beta) may improve the diagnostic accuracy of those scores and may provide additional information regarding the prognosis of patients suffering from CAP [4–7].

Human adrenomedullin (ADM), a 52-amino acid peptide, is a member of the calcitonin peptide family [8]. It is widely expressed and intensively synthesized in organisms suffering from severe infection. It is one of the most potent vasodilating agents and functions in immune modulation, antibiosis and metabolic regulation [9–12]. ADM immediately binds to receptors near the site of its production and has a short half-life [13]. The more stable mid-regional (MR) fragment of the ADM precursor is directly reflective of the level of the rapidly degraded active ADM peptide [14]. Clinically, MR-proADM is commonly used due to its better technical viability than that of ADM. In addition, its level may be indicative of the severity of infection. Increasing evidence has shown that MR-proADM is a superior biomarker compared with others (such as procalcitonin and soluble triggering receptor expressed on myeloid cells-1) for prognostic purposes in sepsis [15, 16], as well as CAP [17]. Thus, we performed this meta-analysis to systematically and quantitatively analyze all available publications that have assessed the prognostic accuracy of the MR-proADM level in CAP patients to draw a firm conclusion from these studies.

## Methods

### Search strategy and selection criteria

Two investigators, Liu D and Xie LX, independently performed the search and assessed the studies. Any disagreement was resolved by consulting with a third investigator (Xie LX). We searched PubMed, Embase, Web of Knowledge and the Cochrane Library. The search terms were as follows: (adrenomedullin or ADM or proADM or "midregional proadrenomedullin" or MR-proADM or proadrenomedullin) and ("respiratory tract infection" or "respiratory infection" or "pneumonia" or "community-acquired pneumonia" or CAP). An example of the search details is presented in Appendix 1. We included articles written in English and Spanish, and no publication date restrictions were applied in the search.

Eligible studies had to have a well-defined reference standard for patients diagnosed with CAP. They had to collect data on MR-proADM levels in adult patients (>18 years old) with mortality or complications from CAP and provide sufficient data for construction of a 2 × 2 contingency table based on the results. Low risk was defined by PSI score classes I to III and CURB-65 score class 1, and high risk was defined by PSI score classes IV–V and CURB-65 score classes 2–5, according to previous criteria [18, 19]. For studies providing multiple MR-proADM cut-off levels for prognostic accuracy, the data presenting the maximum overall accuracy were selected. All published studies had obtained ethics approval and consent for publication of these data. Ethics approval was not sought as this systematic review which synthesized the public data.

### Data extraction and quality assessment

Two investigators, Liu D and Xie LX, independently extracted data and assessed the quality of the included studies. Any conflicts were resolved by consulting with a third investigator. The following data were extracted from the original studies: the name of the first author, publication year, country of origin, study design, clinical setting, assay manufacturer, sample size, endpoints, percentage of high-risk patients according to the PSI score or CURB-65 score, prevalence of mortality or complications, and MR-proADM cut-off level, and the true positive (TP), false positive (FP), false negative (FN), true negative (TN), sensitivity (SEN) and specificity (SPE) of the data. We contacted the corresponding authors if the data were not presented or needed clarification. We evaluated the quality of the included studies according to the Quality Assessment of Diagnostic Accuracy Studies (QUADAS-2) checklist [20] for diagnostic studies. Risk of bias was judged as “low”, “high” or “unclear”.

### Statistical analysis

We chose the MIDAS module of STATA software, version 12.0 (Stata Corporation, College Station, TX) and Meta-Disc 1.4 (XI Cochrane Colloquium, Barcelona, Spain) to perform statistical analyses. TP, FP, FN, and TN were tabulated based on the MR-proADM levels and all-cause mortality in CAP. We used relative risk (RR) to access the predictive value of MR-proADM based on DerSimonian and Laird’s method [21]. The Q-test and *I*^2^ index were conducted to assess inter-study heterogeneity [22, 23]. A *P* value of less than 0.05 was considered statistically significant. Values of 25, 50 and 75 % for the *I*^2^ test represented low, medium and high heterogeneity, respectively [24]. If the *I*^2^ values were less than 50 %, then the fixed-effects model was used; otherwise, the random-effects model was used to analyze the data.

The presence of a threshold effect on the prognostic accuracy of MR-proADM in CAP was evaluated with the Spearman correlation coefficient between the logits of sensitivity and specificity. If no threshold effect existed, then a bivariate random-effects regression model [25] was used to calculate the pooled sensitivity (SEN), specificity (SPE), diagnostic odds ratio (DOR), positive likelihood ratio (PLR), and negative likelihood ratio (NLR). If a threshold effect existed, then we only constructed a summary receiver operator characteristic (SROC) curve by plotting the individual and summary values of sensitivity and specificity to access overall diagnostic accuracy [26].

Univariate meta-regression and subgroup analyses were performed to examine the sources of potential heterogeneity in SEN and SPE. The covariates included the following variables: Consecutive (if studies recruited patients consecutively), Prevalence (prevalence of mortality < 10 % or ≥ 10 %), Sample size (sample size < 500 or ≥ 500), and Blinded (if clinicians influenced patients’ outcomes without knowledge of the MR-proADM levels).

## Results

**Fig. 1:**
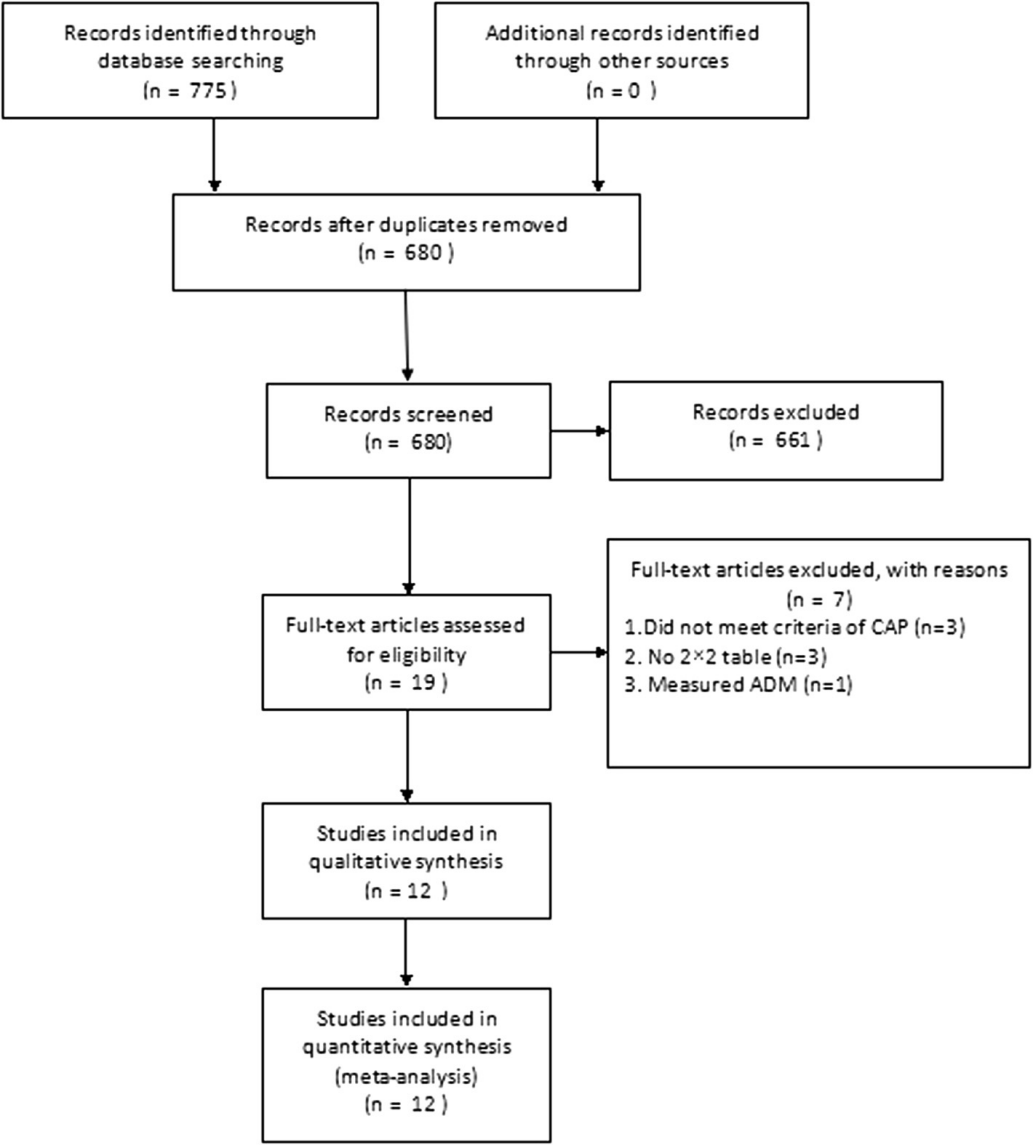
Flow chart of the study selection process

Our database search resulted in the retrieval of 775 articles, of which 746 were eliminated for various reasons related to the title and abstract, leaving 19 studies that were scrutinized by full-text reviews. Among these 19 studies, 1 study measured the level of ADM, 3 did not provide sufficient information to construct a 2 × 2 table, and 3 included ineligible patients (not CAP patients). Ultimately, 12 studies [27–38] fulfilled our eligibility criteria and were included (Fig. 1). The characteristics of the included studies are listed in Table 1.

**Table 1:** Characteristics of included studies

Author	Year	Country	Study design	Clinical setting	Endpoint	Sample size (n)	Male (%)	Age	CURB65 % (2–5)	PSI % (IV–V)	Prevalence of mortality (%)	Cut-off (nmol/L)	TP	FP	FN	TN	Sen (95 % CI)	Spe (95 % CI)
Albrich [27]	2011	Switzerland	MRCT + CR	ED	30 day mortality	925	-	-	53.8	NA	5.41	1.5	36	295	14	580	72	66.3
					Adverse events						14.5	0.75	256	475	11	183	95.9	27.8
Bello [28]	2012	Spain	PR + CR	ED	30 day mortality	224	61	73 (63–80)	59.2	61	5.8	1.066	12	68	1	143	92.3	67.46
					Complications						64	0.833	98	26	48	52	67.35	66.23
Christ-Crain [29]	2006	Switzerland	PR + CR	ED	6.9 ± 1.9 weeks	302	61.9	69.6 ± 17.0	-	60.2	12.6	1.8	30	74	8	190	80	72
Courtais [30]	2013	France	PR + CR	ED	30 day mortality	109	65.1	71.1 (53–84)	-	56	8.26	1.8	7	25	2	75	77.8	75
Huang [31]	2009	America	MPR	ED	30 day mortality	1653	52	65.0 ± 18.5	-	39	6.4	1.3	72	418	34	1129	68	73
Kolditz [32]	2012	Germany	PR + CR	ED	ICU admission or 7 day mortality	51	49	72 (41–80)	-	-	17.6	1.05	6	6	3	36	67	85
Kruger [33]	2009	Germany	RCT + CR	ED	28 day mortality	728	59.1	59 ± 18.2	-	-	2.5	0.959	14	168	4	542	77.8	76.3
Julian-Jimenez [34]	2014	Spain	PR + CR	ED	30 day mortality	127	74	65.8 ± 20.02	55.9	55.1	10.3	1.85	11	21	2	93	84.6	81.4
Suberviola [35]	2012	Spain	PR	ICU	In-hospital mortality	49	67.3	59.4 ± 13.4	-	-	35	4.86	9	5	8	27	53	84
Lacoma [36]	2014	Spain	PR	HW	Complications	85	69.4	-	48.2	61.2	10.6	1.5	6	26	3	50	66.7	65.8
Bereciartua Urbieta [37]	2011	Spain	PR	ED	Unfavorable outcome	250	31.6	71.1	-	-	33.2	1.2	66	78	17	89	80	53
Renaud [38]	2012	America/Spain/France	MRCT + CR	ICU	SCAP	877	58.8	73 (59–83)	-	-	5.6	1.8	49	184	31	613	61.3	76.9

*SCAP* severe community-acquired pneumonia, *ICU* intensive care unit, *ED* emergency department, *HW* hospital ward, *PR* prospective recruitment, *CR* consecutive recruitment, *RR* retrospective recruitment, *RCT* randomized controlled trial, *MPR* multi-center prospective recruitment, *MRCT* multi-center random controlled trial, *TP* true positive, *FP* false positive, *TN* true negative, *FN* false negative, *SEN* sensitivity, *SPE* specificity, *CI* confidence interval

### Characteristics of included studies

The included studies were published from 2006 to 2014. Ten studies [27–33, 35, 36, 38] were published in English, and two [34, 37] were in Spanish. All of the studies were prospective cohorts, and three [27, 31, 38] were multiple-center trials. Three of the studies [27, 33, 38] selected patients from a randomized clinical trial. All of the studies were conducted in Europe. The mean patient ages varied from 59 to 73 years, and the proportion of men ranged from 31.6 to 74. The prevalence of mortality ranged from 2.5 to 35. The prevalence of complications ranged from 5.6 to 64. The studies were performed in EDs [27–34, 37], ICUs [35, 38] and HWs [36]. The primary endpoint was development of complications [27, 28, 36–38], and the secondary endpoint was mortality [27–35]. In all of the studies, MR-proADM was detected by an automated immunofluorescence assay (BRAHMS MR-proADM KRYPTOR, BRAHMS GmbH, Hennigsdorf, Germany) [39].

### Study quality and publication bias

The quality of the included studies is shown in Appendix 2. Deek’s Funnel Plot is presented in Additional file 1: Figure S1.

### Data synthesis and meta-analysis

#### Analysis of the association of MR-proADM with development of complications

Five studies [27, 28, 36–38] with a total of 2361 patients were included in this group. However, a significant threshold effect was observed (Spearman correlation coefficient = 0.900; *P* = 0.037). Therefore, we only calculated the overall area under the SROC curve (AUC), which was 0.74 (95 % CI: 0.70–0.78) (Additional file 2: Figure S2.).

#### Analysis of the effect of MR-proADM on mortality

Nine studies [27–39] with a total of 4119 patients were included in this group. All of them showed that an elevated MR-proADM level was associated with higher risk of death from CAP. Because the heterogeneity between studies was 21.7 %, the fixed-effects model was used. The pooled RR was 5.83 (95 % CI 4.53–7.52) (Additional file 3: Figure S3.).

**Fig. 2:**
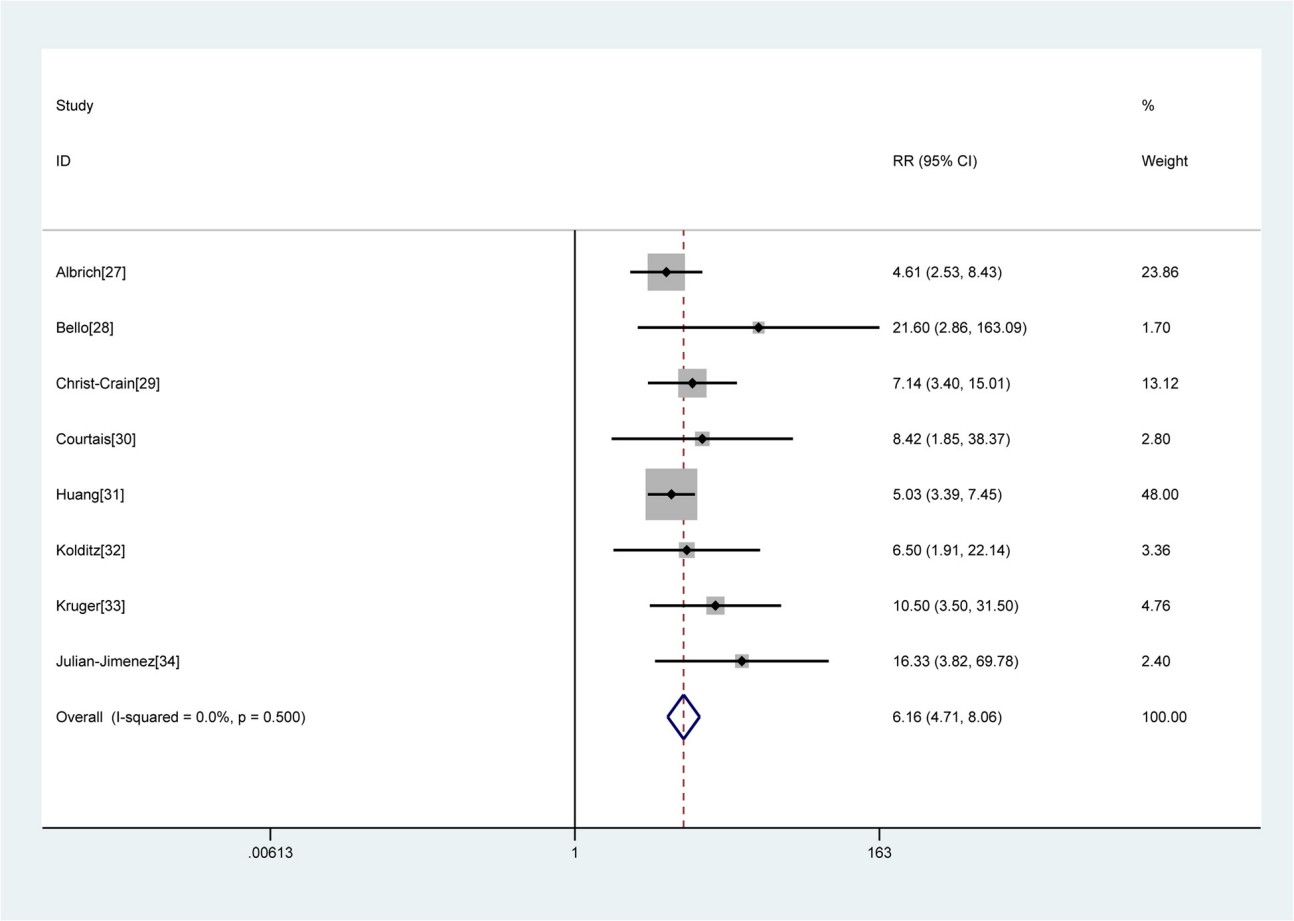
Forest plot of association of MR-proADM to predict mortality in CAP. The overall pooled RR was 6.16 (95 % CI, 4.71–8.06)

One study [35] analyzed CAP patients in an ICU with a relatively high mortality rate. We found that this study had a more heterogeneous population compared with the other included studies. Thus, we excluded it and focused on the association of the MR-proADM level with short-term mortality in ED patients. Because the heterogeneity between studies was 0.0 %, a fixed-effects model was used to pool RR. For the ED patients, an elevated MR-proADM level was associated with an increased risk of short-term mortality (RR 6.16, 95 % CI 4.71–8.06) (Fig. 2).

**Fig. 3:**
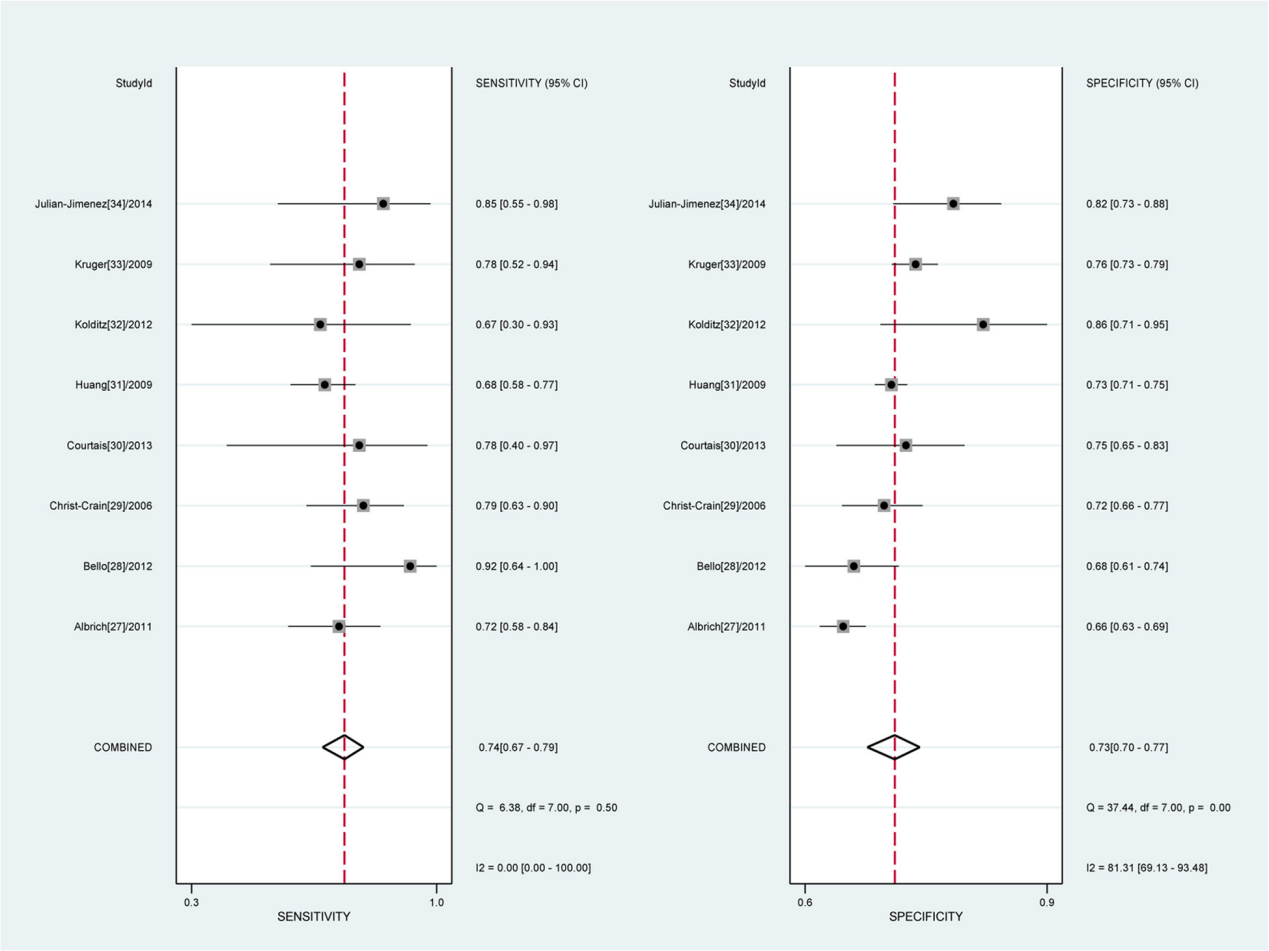
Forest plot of the sensitivity and specificity of MR-proADM to predict mortality in CAP. The pooled sensitivity and specificity were 0.74 (95 % CI: 0.67–0.79) and 0.73 (95 % CI: 0.70–0.77), respectively

**Fig. 4:**
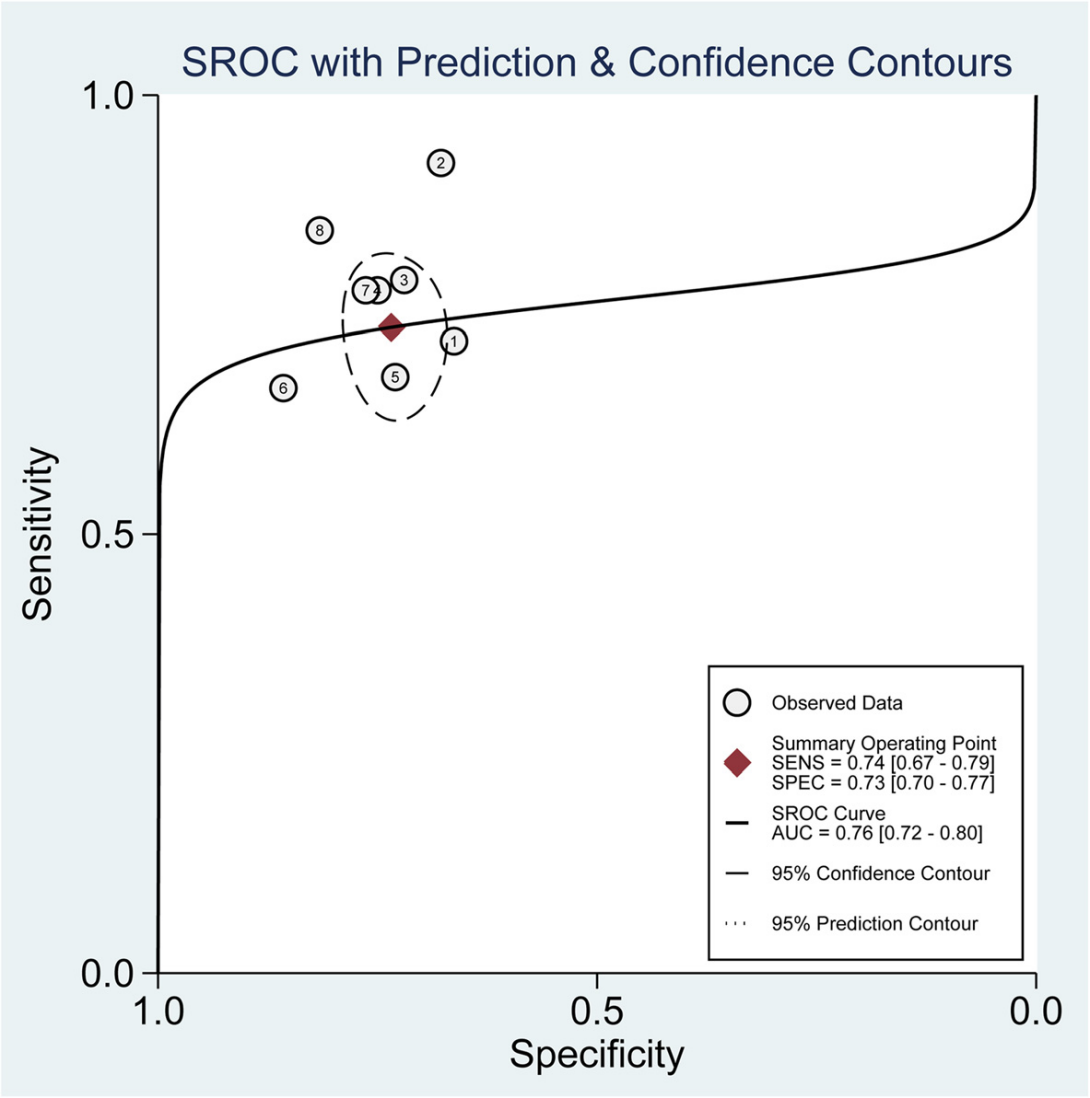
Summary receiver operating characteristic (SROC) curve for the included studies. The numbers in the circle refer to the included studies; Line = regression; the overall area under the SROC curve was 0.76 (95 % CI, 0.72–0.80)

We observed no statistically significant differences in threshold effects (Spearman correlation coefficient = 0.252; *P* = 0.548). Thus, the bivariate random-effects regression model was used to perform meta-analysis of diagnostic test accuracy to evaluate the overall sensitivity and specificity of MR-proADM for predicting mortality in CAP. The pooled sensitivity and specificity were 0.74 (95 % CI: 0.67–0.79) and 0.73 (95 % CI: 0.70–0.77), respectively (Fig. 3). The positive likelihood ratio (PLR) and negative likelihood ratio (NLR) were 2.8 (95 % CI, 2.3–3.3) and 0.36 (95 % CI, 0.29–0.45), respectively. The diagnostic odds ratio (DOR) was 8 (95 % CI, 5–11). The overall area under the SROC curve was 0.76 (95 % CI, 0.72–0.80) (Fig. 4), indicating moderate diagnostic accuracy. The mean cut-off MR-proADM level for predicting mortality in CAP was 1.416 ng/ml (IQR 0.959–1.85).

**Fig. 5:**
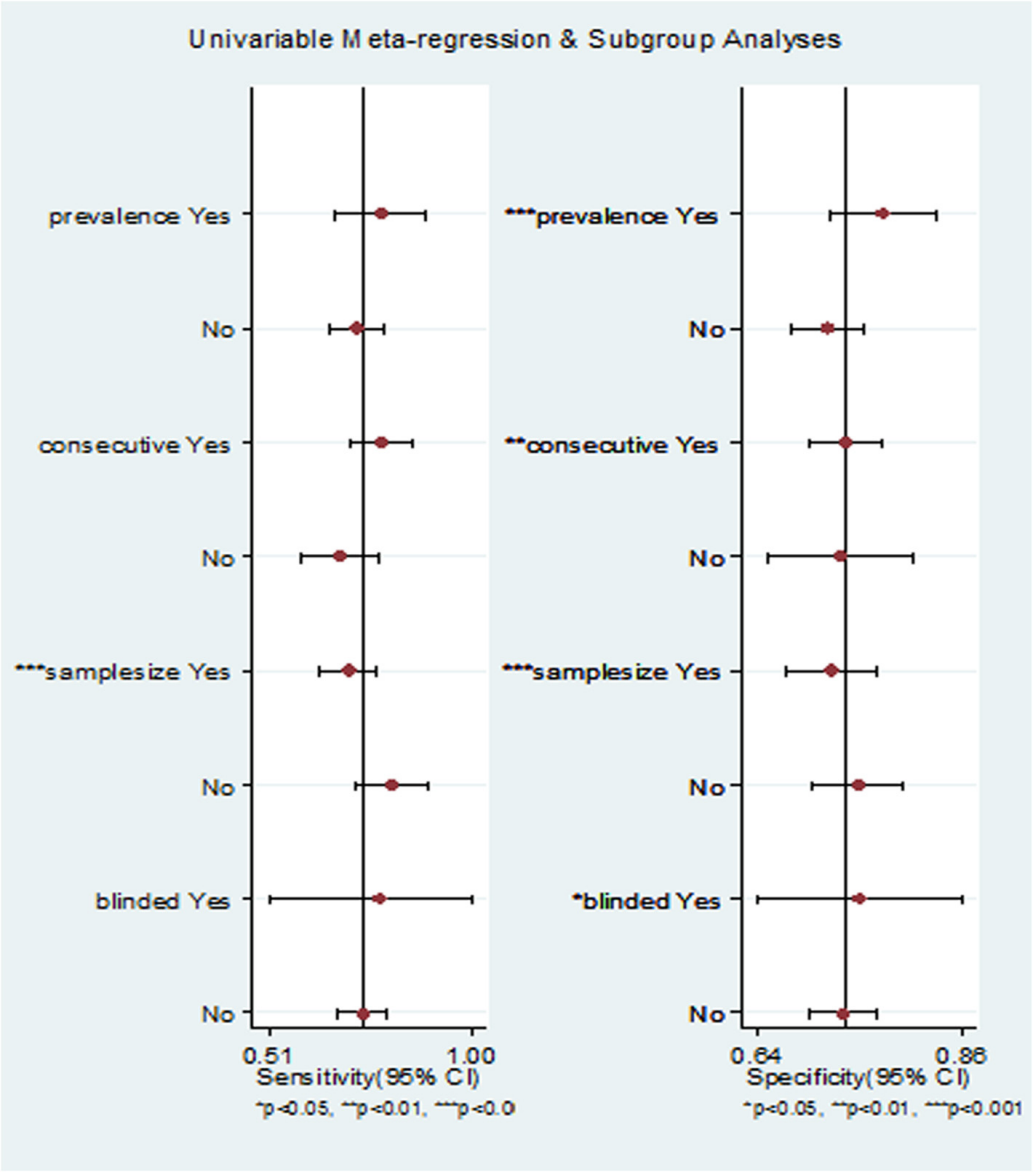
Univariate meta-regression and subgroup analyses (Sample size was significantly associated with the heterogeneity of SEN, whereas Consecutive, Sample size, Prevalence and Blinded were significantly associated with the heterogeneity of SPE)

The overall *I*^*2*^ value for the bivariate model was 0.0 % (95 % CI 0–100). The *I*^*2*^ values for the pooled SEN and SPE were 0.00 % (95 % CI 0–100) and 81.31 % (95 % CI 69.13–93.48), respectively. Univariate meta-regression and subgroup analyses were performed to examine the sources of potential heterogeneity in SEN and SPE. The covariates included the following: Consecutive (if studies recruited patients consecutively), Prevalence (prevalence < 10 % or ≥ 10 %), Sample size (sample size < 500 or ≥ 500), and Blinded (if clinicians influenced patients’ outcomes without knowledge of the MR-proADM levels). The results showed that sample size was significantly associated with the heterogeneity of SEN, whereas the covariates Consecutive, Sample size, Prevalence and Blinded were significantly associated with the heterogeneity of SPE (Fig. 5). We performed subgroup analysis according to the different prevalence rates of mortality. Three of the studies [29, 32, 34] had a prevalence of over 10 %, with pooled sensitivity and specificity values of 0.78 (95 % CI: 0.67–0.89) and 0.78 (95 % CI: 0.72–0.84), respectively. In addition, five of the studies [27, 28, 30, 31, 33] had a prevalence of less than 10 %, with pooled sensitivity and specificity values of 0.72 (95 % CI: 0.66–0.78) and 0.72 (95 % CI: 0.68–0.75), respectively.

## Discussion

In this meta-analysis, we restricted our scope to patients in the ED. We aimed to help clinicians to select the most appropriate care setting for CAP patients in the ED, including outpatient treatment, admission to a hospital ward, or admission to an intensive care unit. We first found that an elevated MR-proADM level was significantly associated with an increased risk of mortality in the ED patients with CAP. Clinical scores are recommended for clinical decision-making in the evaluation of CAP patients [40]. However, many studies have found that these clinical scores are not exempt from false-positive and false-negative results and are not ideal for clinical use. Many patients are misclassified into the high-risk classes IV and V according to the PSI score [41]. Meta-analysis [42] has shown that the CURB-65 score has a sensitivity of only 0.62 for predicting mortality in CAP. Marrie et al. [43] have demonstrated that a considerable number of CAP patients identified as high risk based on a PSI level of IV or V can be treated safely as outpatients, with subsequent low mortality. Our study evaluated the prognostic value of ProADM in CAP and revealed that the positive likelihood ratio (PLR) and negative likelihood ratio (NLR) were 2.8 (95 % CI, 2.3–3.3) and 0.36 (95 % CI, 0.29–0.45), respectively. These results indicate that MR-proADM is more clinically useful than any of the risk scores previously identified in another meta-analysis [42].

Because the included studies used different cut-off values, simply pooling data from each trial may have contributed to bias in the meta-analysis results. Thus, in our meta-analysis, we conducted statistical analysis to ensure the eligibility and reliability of our results before tabulating the data from each trial. We used Meta-Disc to evaluate the Spearman correlation coefficient. We calculated the RR, SEN, SPE, DOR, PLR, and NLR for each trial based on the premise that there were no significant differences in the threshold effects (*P* > 0.05), indicating that the heterogeneity attributed to the use of different cut-off values was acceptable. Thus, our results are more accurate than those of other diagnostic and prognostic meta-analyses.

Our study has several limitations. First, all of the studies included in our meta-analysis were conducted in Europe. Thus, our results are restricted to Europeans. Second, Deek’s funnel plot revealed the existence of potential publication bias. Third, we could not determine the optimized cut-off value because we failed to obtain the raw data from each original article to construct an ROC curve. We attempted to contact the corresponding authors to obtain the data, but it was difficult to acquire the ProADM levels of the patients in each trial. Albrich et al. [27] have examined the ProADM level and CURB65 score combined and have found that the risk of unfavorable outcome is low for patients with a CURB65 score of 0–1 and a ProADM level of ≤0.75 nmol/l, intermediate for patients with a CURB65 score of 2 and a ProADM level of ≤1.5 nmol/l or a CURB class of 0–1 and a ProADM level of between 0.75 and 1.5 nmol/L, and high for all other patients. Another study [44] has also focused on the prognosis of CAP patients using these ProADM cut-off levels. All in all, further studies are warranted to define the proper cut-off level for clinical use.

CAP is a complex pathophysiological process rather than a specific syndrome. Thus far, no ideal biomarker or clinical score has shown sufficient sensitivity and specificity for clinical utility to predict death in CAP. Clinicians need to comprehensively evaluate individual conditions. Future research should highlight incorporation of MR-proADM into an overall assessment of CAP prognosis in combination with other clinical indexes instead of focusing on adopting a biomarker-based or score-based approach to predicting mortality. Additionally, future studies should be conducted specifically on patients with different conditions (e.g., different CAP severities or different types of infection) to help optimize therapeutic decisions for individual patients.

## Conclusions

Our study has demonstrated that MR-proADM is predictive of increased complications and a higher mortality rate in patients suffering from CAP. Further studies are warranted to clarify the prognostic accuracy of MR-proADM in conjunction with severity scores or other biomarkers and to determine an optimal cut-off level.

## Abbreviations

CAP, community-acquired pneumonia; CI, confidence interval; CR, consecutively recruitment; DOR, diagnostic odds ratio; ED, emergency department; FN, false negative; FP, false positive; LR, likelihood ratio; MPR, multiple-centre prospectively recruitment; MRCT, multiple-centre random control trail; MR-proADM, midregional proadrenomedullin; NLR, negative likelihood ratio; PLR, positive likelihood ratio; PR, prospectively recruitment; QUADAS, quality assessment of diagnostic accuracy studies; RCT, random control trail; RR, risk ratio; SEN, sensitivity; SPE, specificity; SROC, summary receiver operator characteristic; TN, true negative; TP, true positive.

## Appendix 1

### Example of search strategy for PubMed

(("adrenomedullin"[MeSH Terms] OR "adrenomedullin"[All Fields]) OR ("Rev ADM"[Journal] OR "adm"[All Fields] OR "Rev ADM"[Journal] OR "adm"[All Fields] OR "ADM"[Journal] OR "adm"[All Fields] OR "ADM"[Journal] OR "adm"[All Fields]) OR proADM[All Fields] OR "midregional proadrenomedullin"[All Fields] OR MR-proADM[All Fields] OR ("proadrenomedullin"[Supplementary Concept] OR "proadrenomedullin"[All Fields])) AND ("respiratory tract infection"[All Fields] OR "respiratory infection"[All Fields] OR "pneumonia"[All Fields] OR "community-acquired pneumonia"[All Fields] OR CAP[All Fields])

## Appendix 2

**Table 2:** QUADAS-2 results of included studies

Study	Risk of bias				Applicability concerns		
	Patient selection	Index test	Reference standard	Flow and timing	Patient selection	Index test	Reference standard
Albrich [27]	L	L	L	L	L	L	L
Bello [28]	L	L	L	L	L	L	L
Christ-Crain [29]	L	L	L	L	L	L	L
Courtais [30]	L	L	L	L	L	L	L
Huang [31]	H	L	L	L	H	L	L
Kolditz [32]	L	L	L	L	L	L	L
Kruger [33]	L	L	L	L	L	L	L
Julian-Jimenez [34]	L	L	L	L	L	L	L
Suberviola [35]	H	L	L	L	H	L	L
Lacoma [36]	H	L	L	L	H	L	L
Bereciartua Urbieta [37]	H	L	L	L	H	L	L
Renaud [38]	L	L	L	L	L	L	L

*L* low risk, *H* high risk, *U* unclear risk

## Additional files

Additional file 1:
**Figure S1.** Deek’s funnel plot. Deek’s funnel plot asymmetry test for publication bias (A. for development of complications; B. for prediction of mortality). (TIF 755 kb) Additional file 2:
**Figure S2.** SROC curve of the included studies. The numbers in the circle refer to the included studies; Line = regression; the overall area under the SROC curve was 0.74 (95 % CI: 0.70–0.78). (TIF 241 kb) Additional file 3:
**Figure S3.** An elevated MR-proADM level was associated with a higher risk of death in CAP. The pooled RR was 5.83 (95 % CI 4.53–7.52). (TIF 215 kb)
