# Supplementary figures and images for: Prognostic value of mid-regional pro-adrenomedullin (MR-proADM) in patients with community-acquired pneumonia: a systematic review and meta-analysis
Source: BMC Infect Dis. 2016 May 26;16:232. doi: 10.1186/s12879-016-1566-3 (PMC4881068; doi:10.1186/s12879-016-1566-3)

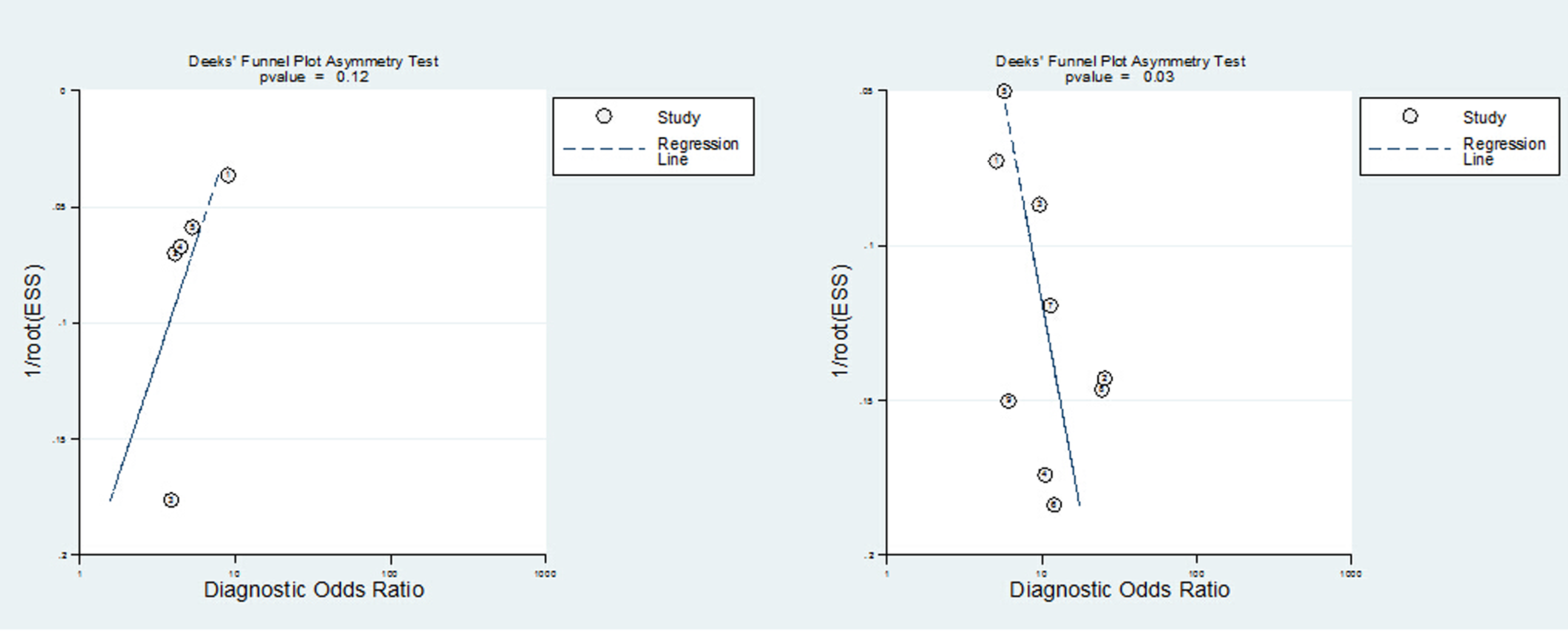

Supplement: Additional file 1: — Figure S1. Deek’s funnel plot. Deek’s funnel plot asymmetry test for publication bias (A. for development of complications; B. for prediction of mortality). (TIF 755 kb) [file 12879_2016_1566_MOESM1_ESM.tif]

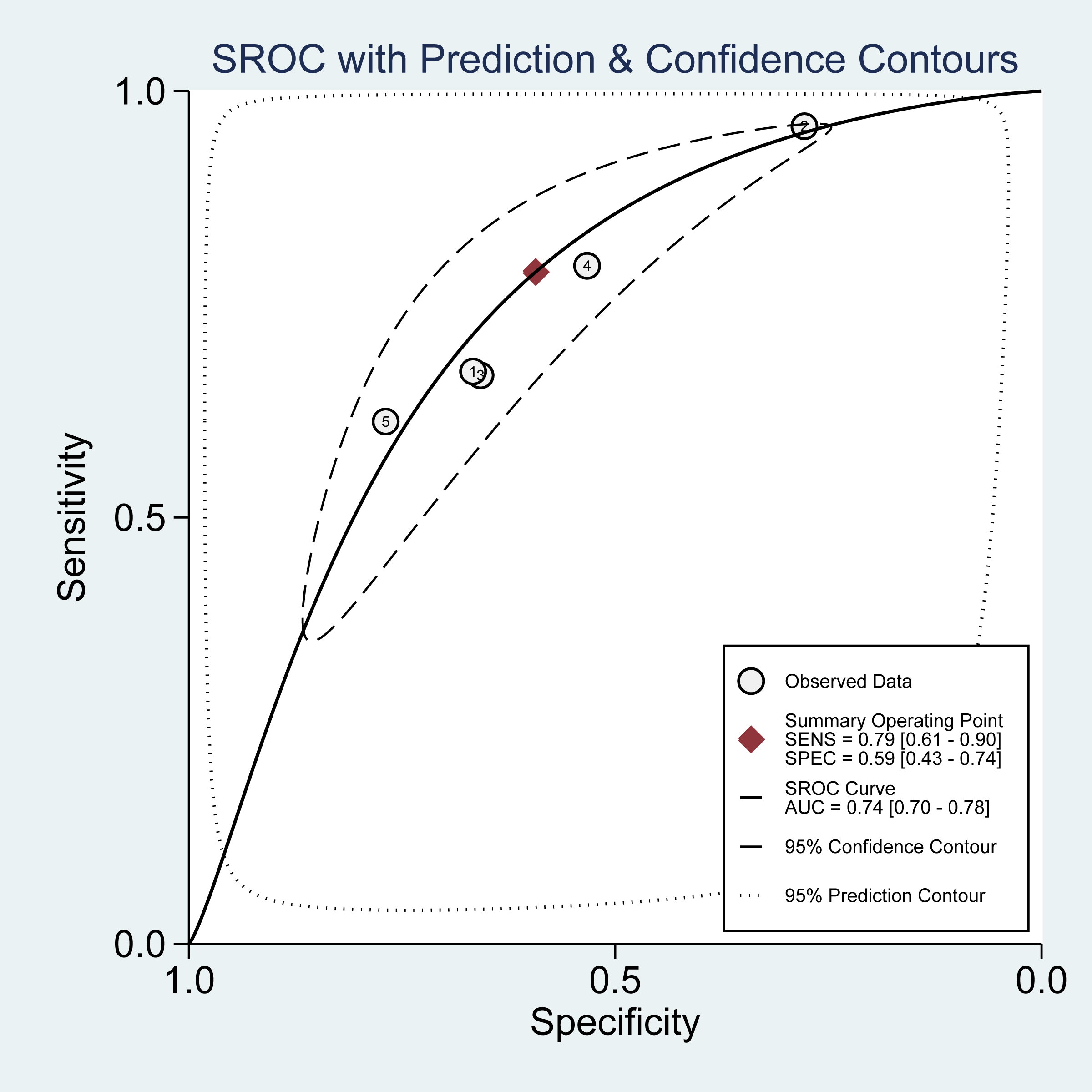

Supplement: Additional file 2: — Figure S2. SROC curve of the included studies. The numbers in the circle refer to the included studies; Line = regression; the overall area under the SROC curve was 0.74 (95 % CI: 0.70–0.78). (TIF 241 kb) [file 12879_2016_1566_MOESM2_ESM.tif]

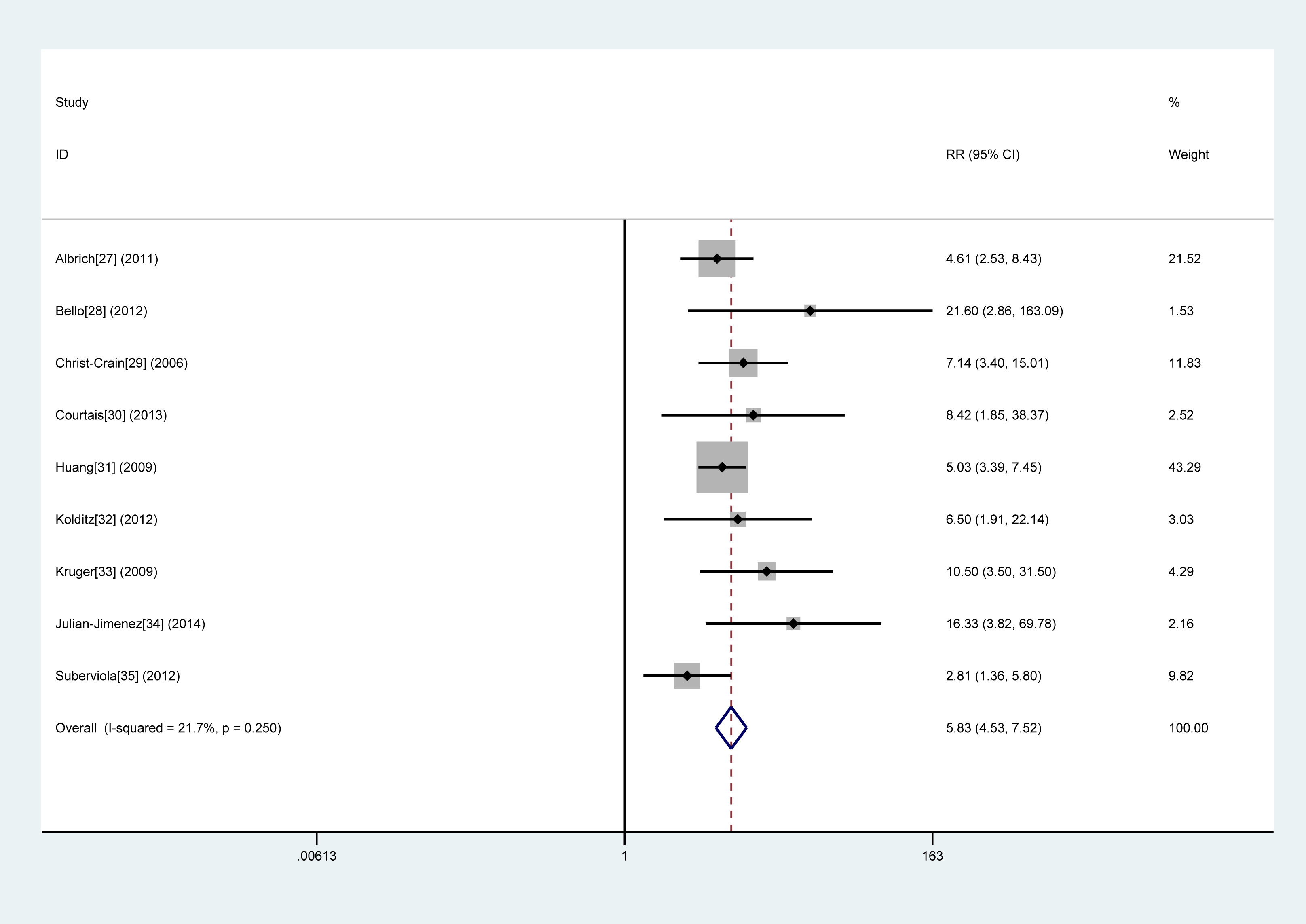

Supplement: Additional file 3: — Figure S3. An elevated MR-proADM level was associated with a higher risk of death in CAP. The pooled RR was 5.83 (95 % CI 4.53–7.52). (TIF 215 kb) [file 12879_2016_1566_MOESM3_ESM.tif]
